# Prevalence and predictive factors for depressive symptoms among medical students in Germany – a cross-sectional study

**DOI:** 10.3205/zma001534

**Published:** 2022-02-15

**Authors:** Lilith Pukas, Nadja Rabkow, Lea Keuch, Emilia Ehring, Stephan Fuchs, Dietrich Stoevesandt, Alexandra Sapalidis, Angelina Pelzer, Carolin Rehnisch, Stefan Watzke

**Affiliations:** 1 University Hospital Halle (Saale), University Clinic and Outpatient Clinic for Psychiatry, Psychotherapy and Psychosomatics, Halle (Saale), Germany; 2 University Hospital Halle (Saale), Institute of General Medicine, Halle (Saale), Germany; 3 Martin Luther University of Halle-Wittenberg, Medical Faculty, Dorothea Erxleben Learning Centre, Halle (Saale), Germany

**Keywords:** depression, medical students, risk factors, protective factors, prevalence, mental health, prevention, intervention targets, predictive factors

## Abstract

**Background: **Elevated levels of depressive symptoms among medical students have been the subject of international and national research before, yet associated risk factors and protective factors are to be determined. This study aims to show the burdens of depression at different stages of academic medical education with a special emphasis on correlated risk factors and protective factors.

**Methods: **A total number of n=1103 medical students of a middle-sized German university were sampled and surveyed regarding depressive symptoms and correlating factors. The assessment of potential depressive symptoms was based on the BDI-II. Correlating factors were surveyed through a self-designed questionnaire consisting of possible cofactors for depressive symptoms based on established scientific literature.

**Results: **Survey response rate was 90.2% (1103/1223). The prevalence of depressive symptoms was 11% for mild, 5.6% for moderate and 2.4% for severe symptoms. The sample’s most prevalent risk factors were feeling unable to confide one’s own worries to someone else (88%); and experiencing a lack of time for partner, friends and family (77%) or hobbies (76%). Significant predictors for depressive symptoms were neuroticism above all, insufficient emotional support, eating irregular meals, use of medication or drugs to calm down, and mental overload. Factors associated with less depressive symptoms could be identified as: spending time with partner, friends, family, hobbies and exercise; and confiding worries to classmates.

**Conclusions:** Every fifth medical student surveyed reported at least mild depressive symptoms. The majority of the surveyed medical students felt unable to confide their worries to someone else and lamented not having enough time for social interaction with peers, family and hobbies. Certain personality traits – such as neuroticism – and insufficient emotional support showed to play important roles in making medical students more prone to developing depressive symptoms. Based on this research, control of the surveyed cofactors associated with depressive symptoms and possible intervention programs targeted to these are proposed to be a key subject of further research.

## Zusammenfassung

**Hintergrund: **Erhöhte depressive Symptome bei Medizinstudierenden waren bereits weitgehender Gegenstand internationaler und nationaler Forschung, jedoch sind assoziierte Risikofaktoren und Schutzfaktoren bis dato ungenügend ermittelt worden. Ziel dieser Studie ist es, die Belastungen durch depressive Symptome in verschiedenen Stadien der akademischen medizinischen Ausbildung mit besonderem Schwerpunkt auf korrelierte Risikofaktoren und Schutzfaktoren aufzuzeigen.

**Methoden: **Insgesamt wurden n=1103 Medizinstudierende einer mittelgroßen deutschen Universität in die Stichprobe aufgenommen und zu depressiven Symptomen und korrelierenden Faktoren befragt. Die Beurteilung möglicher depressiver Symptome erfolgte nach dem BDI-II. Korrelierende Faktoren wurden durch einen selbst entworfenen Fragebogen erhoben, welcher auf der Grundlage etablierter wissenschaftlicher Literatur mögliche Kofaktoren für depressive Symptome enthielt.

**Ergebnisse: **Die Rücklaufquote der Umfrage betrug 90,2% (1103/1223). Die Prävalenz depressiver Symptome betrug 11% für leichte, 5,6% für mittelschwere und 2,4% für schwere Symptome. Die häufigsten Risikofaktoren der Stichprobe waren der Umstand, die eigenen Sorgen niemand anderem anvertrauen zu können (88%); und Mangel an Zeit für Partner, Freunde und Familie (77%) oder Hobbies (76%). Signifikante Prädiktoren für depressive Symptome waren vor allem Neurotizismus, als unzureichend erlebte emotionale Unterstützung, die Einnahme unregelmäßige Mahlzeiten, Einnahme von Medikamenten oder Drogen zur Selbstberuhigung und subjektives Überforderungserleben. Faktoren, die mit weniger depressiven Symptomen verbunden waren, konnten identifiziert werden als: Zeit mit (Ehe-)Partner, Freunden, Familie, Hobbys und Sport verbringen; und Kommilitonen eigene Sorgen anvertrauen.

**Schlussfolgerungen: **Jeder fünfte befragte Medizinstudierende berichtete von mindestens leichten depressiven Symptomen. Die Mehrheit der befragten Medizinstudierenden fühlte sich nicht in der Lage, ihre Sorgen einer anderen Person anzuvertrauen und gaben an, dass sie nicht genügend Zeit für soziale Interaktionen mit Gleichaltrigen, Familie und Hobbys hätten. Neurotizismus und als unzureichend erlebte emotionale Unterstützung erwiesen sich als eng mit depressiven Symptomen korreliert. Basierend auf dieser Arbeit werden wissenschaftliche Forschungsansätze im Bereich der Kontrolle der untersuchten, mit depressiven Symptomen assoziierten Kofaktoren, sowie die Erforschung möglicher an diesen Stellen ansetzender Interventionsprogramme für einen Schwerpunkt weiterer Untersuchung vorgeschlagen.

## 1. Introduction

A meta-analysis investigating the prevalence of depressive symptoms among medical students [1] reported that one in three medical students worldwide may be affected with elevated levels of depressive symptoms (27.2%; 95% Confidence Interval, 24.7%-29.9%). These results are widely interpreted as indicating a higher risk of Major Depression in medical students internationally [2], [3]. Medical students themselves play a minor role in healthcare. owever, postgraduation their role shifts to an essential one, as they become fully qualified practicing doctors. Depressive symptoms – exempli gratia suicidal thoughts [4] – and perceived study stress in medical school have been related to mental health problems in postgraduates before [5], [6]. Similarly, medical training was identified as a negative influence on the mental health of medical residents in the United States [7]. Not only Major Depression poses a significant economic burden to society [8], depressed doctors could be at higher risk for making medical errors [9], [10]. Ultimately, this results in poorer patient care in addition to personal and economic harm. 

In an international context, the prevalence and severity of depressive symptoms in medical students has been widely researched. Six of the 183 studies analysed by Rotenstein et al. have been conducted in Germany [11], [12], [13], [14], [15], [16], [17]. Additional research was identified through intensive search of GoogleScholar, Pubmed and Medline [16], [18], [19]. However, in-depth research related to possible correlating factors for medical students’ depressive symptoms in Germany was inconclusive and scarce [13], [20]. Additionally, most available studies comparatively examined small samples, recorded low response rates and focused on isolated classes of risk factors. In this study, a multitude of potential cofactors will be surveyed and discussed. 

This study aims to evaluate the prevalence and severity of depressive symptoms among medical students. Its strength lies in the evaluation of large study samples covering students from major time points during the course of their medical studies combined with an excellent response rate. This allows us to showcase a valid and differentiated assessment of a wide range of possible cofactors for depressive symptoms. Our research could be a base for future exploration of targeted, low-threshold services to aid affected students after thorough scientific control of the surveyed cofactors mentioned in this study. 

To screen for potential correlating factors, this study used a self-designed questionnaire. The screening instrument used for the survey of depressive symptoms was the BDI-II by Beck et al. [21] as it has been well established in clinical and non-clinical settings.

## 2. Methods

### 2.1. Data set 

Data was collected from (n=1103) medical students attending a middle-sized German university. The students were surveyed from 10/2017 to 10/2018. 

#### 2.2. Study design

Medical studies in Germany have a regular duration of 12 semesters and are terminated by a state examination degree. The students are obliged to pass summative exams – after the 4^th^, 10^th^ and 12^th^ semester – before being allowed to progress to the next phase. Content-wise, the medical curriculum is divided into “pre-clinical” (1^st^-4^th^ semesters) and “clinical” section (5^th^-12^th^ semesters). 

Students from times with critical importance for the course of medical studies were asked to participate. Those surveyed were freshmen (1^st^ semester), students prior the preclinical summative exam (4^th^ semester), post the preclinical exam (5^th^ semester) and prior the clinical exam (9^th^ & 10^th^ semesters). Surveys were conducted in small seminar groups. The inclusion of students attending the 11^th^ & 12^th^ semesters was not attempted as the programme’s structure as a sole clinical rotation made it difficult to guarantee sufficient response rates. Additionally, comparison between students actively attending university classes and students solely attending a clinical rotation could have been invalid. 

An ethical approval of the study methods was obtained. After signing an informed consent form, potential subjects were handed a questionnaire during compulsory seminars either at the beginning or at the end of a running academic term. One survey per respective academic term took place. Subjects were asked to answer the questionnaire’s items in private to guarantee a reliable and independent survey. Time required for the completion of the questionnaire was around 30 minutes.

#### 2.3. Participants

Of 1700 students enrolled at the medical faculty, students from the semesters mentioned above were especially targeted. Hence 64.9% (n=1223) of the whole student cohort were asked to participate. From these, 90,2% (n=1103) students actively participated and were included in this survey. The number of surveyed students was spread evenly among the programme’s “pre-clinical” section (51%) and the “clinical” section (49%). The numbers of students surveyed from 1^st^, 4^th^ and 5^th^ semesters ranged from n=214 to n=350 respectively. The senior year consisted of 9^th^ and 10^th^ semester students which contributed to a total of n=310 subjects. Participants’ ages ranged from 17 to 45 years (Median: 23.1 years, Standard Deviation: 4.0). The female to male ratio was 64.9:35.1 [%f:%m]. The overall response rate for this study was 90.2% (see table 1 (Tab. 1)). 

#### 2.4. Measure 

##### 2.4.1. Independent variables – risk factors and protective factors

To screen for potential correlating factors, this study used a self-designed questionnaire (see attachment 1 ). Besides sociodemographics - for example age and gender – well-researched correlating factors previously associated with depressive symptoms in general were included. Possible risk factors such as *positive family* [22] and own *history of mental illness* [23], *socioeconomic status* [24] as illustrated by highest parental academic attainment, and *stressful life events* [25], [26] (e.g. social relationships, exams, high work load, physical distance from friends and family, financial situation), as well as *prescription and recreational drug abuse* [27], [28], *social isolation* and *loneliness* [29] were collected. Also, the students were surveyed on potential protective factors such as* proactive coping mechanisms* [30], *personal targets* [31], *exercise* [32] or *yoga* [33], *active playing of a musical instrument* [34], [35], *religiosity or spirituality* [36], [37] and *support from friends, family or teachers* [38]. Since *neuroticism* showed to be previously associated with depressive symptoms [39], [40], this personality trait was assessed using the corresponding subscale of 12 items of the fully standardized NEO-FFI. It represents a widely used personality inventory whose assessment results in an objective, reliable and well-validated summary score. The response format was a five-point Likert scale as developed by Costa et al. [41]. There has been no factor adaptation or replication and no changes in item scoring.

##### 2.4.2. Dependent variable – depressive symptoms 

The BDI-II by Beck et al. [21] was used to assess for potential depressive symptoms. The BDI-II has been established in clinical and non-clinical settings. It has favourable psychometric characteristics and discriminates well between severity levels of depression [42]. It is an objective, reliable and valid test procedure [43]. The BDI-II uses 21 items to record the severity of depressive symptoms. It utilizes a self-report, multiple-choice inventory and a multiple-response format with each answer being scored on a 4-point scale ranging from zero to three. The standardized cut-offs indicate the severity of a subject’s depressive symptoms and were scored as originally recommended by Beck et al. [21]. In this study there has been no factor adaptation or replication; the BDI-II items were scored as originally recommended.

#### 2.5. Data analysis 

The statistical analysis was carried out using the software SPSS 25.0. To evaluate the frequency and clinical significance of depressive symptoms, descriptive statistics and determination of BDI-II sum scores’ relative frequencies were used. The sample’s sociodemographic description was based on the descriptive distribution characteristics such as mean, median, standard deviation and range. Since the BDI-II total score was skewed and significantly deviated from normal distribution (Kolmogorov-Smirnoff-Z=.137; p<0.001), non-parametric comparison of subsamples (semester cohorts) was evaluated using Kruskal-Wallis-H (global group comparison) and Mann-Whitney-U (single comparisons). For correlations between risk factors and BDI-II scores, Kendall-tau was calculated. Because multiple bi-variate correlations were calculated, adjusting statistical significance for the number of tests was necessarily discussed. We decided to report all levels of significance and complete the analysis by multivariate linear regression to identify the most influential risk factors for depressive symptoms. Therefore, a stepwise linear multiple regression (p_in_≤.05, p_out_≥.10) was conducted. This also reduced the issue of possible multicollinearity by selecting additional predictors solely when they exhibited high partial correlation with the given criterion. 

## 3. Results

### 3.1. Prevalence of depressive symptoms 

The BDI-II questionnaire showed a high internal consistency (α=.90). The mean BDI-II score among the subjects was 8.32 points (Standard Deviation=7.14). Median was 6, range from 0 to 46 points. The interquartile range was from 3 to 11 points. The surveyed BDI-II mean scores and their translation into levels of depression are recorded in table 2 (Tab. 2). Symptom scores differed globally between subsamples (Kruskal-Wallis-H[df=3] = 14.06; p=0.003). Post-hoc-comparisons (Mann-Whitney-U) showed significant differences between 1^st^ and 4^th^ semester (p=0.011), 5^th^ (p=0.029) and 9^th^/10^th^ semester (p<0.001). Students of the 4^th^ and 5^th^ semester did not differ significantly (p=.0.679), both subsamples, however, differed from subsample 9^th^/10^th^ semester (both p<0.001). 

Highest BDI-II item scores were found for the *changes in sleep patterns, tiredness and fatigue, self-criticism, loss of energy,* and *problems with focus*. Lowest mean scores were found for suicidal thoughts, albeit 11.6% (n=129) of the total sample reported having experienced *suicidal thoughts without direct intention to act.*

#### 3.2. Risk factors and protective factors correlated with depressive symptoms 

For an overview of all correlated factors with BDI-II see attachment 2 . Highest correlations in the total sample were found for: *NEO-FFI neuroticism subscale (internal consistency: α=.88); insufficient emotional support; feeling overwhelmed; performance pressure, loneliness and lack of time for partner, friends or family.* Those correlations were found respectively within all subsamples. The sample’s most prevalent risk factors were:* feeling unable to confide own worries to someone else* (88%) and experiencing a *lack of time for partner, friends and family* (77%) or *hobbies* (76%). 

Factors associated with fewer depressive symptoms could be identified as: *intake of regular meals; amount of time spent with hobbies and exercise; spending time with partner, friends, family and confiding one’s worries to classmates (see attachment 2 ).*

In order to identify risk factors for depressive symptoms with less collinearity, a stepwise multivariate linear regression model including all variables was conducted. This model explained 61.7% of the entire sample’s BDI-II total score’s variance (corrected R^2^). Significant predictors for depressive symptoms were neuroticism above all, and the following items in decreasing order of importance:* insufficient emotional support; eating irregular meals; use of medication or drugs to calm down; feeling overwhelmed* followed by other predictors with less than 5% additional explanation of variance (see table 3 (Tab. 3)). 

## 4. Discussion

Our findings illustrate the high number of students that reported depressive symptoms, but also the high variance of symptom burden. The prevalence of depressive symptoms in Germany’s general population is 9,2% with young adults (age<28) showing slightly elevated depressive symptoms with a prevalence of 11,5% [44]. Normative data on university students of all courses tested by Beck et al. [21] showed a mean BDI-II total score of 12.6 (Standard Deviation: 9.9, n=120). Data of the mean BDI-II score among German law students exhibited a mean value of 11.9 (±8.45) points [45]. These BDI-II scores were slightly higher than our own findings (8.32 (±7.14)). 

Integrating our results onto an international scale, research on n=15,233 American college students [46] of various courses reported a mean BDI-II total score of 9.14 (SD 8.45) – again, it is similar to our findings. It appears that medical students do not exhibit the more severe depressive symptoms, yet in general university students appear to experience depressive symptoms. High-quality research is fundamental to explore the prevalence of depressive symptoms among German university students in general compared to medical students. Approximately 11,6% of our subjects experienced suicidal ideation, which was similar to international [1], [47] and national findings [17]. 

Notably, depressive symptoms were especially pronounced during the 4^th^ and 5^th^ semester and declined towards the end of the course’s duration. One might argue for a rise in resilience among the more advanced medical students, however, the decline in their sub-sample size should not be ignored. Severely depressed medical students may have dropped out of studies and in turn may not have contributed to the results of this survey in terms of a selection bias.

Furthermore, gender has been discussed as an important factor in mental health of medical students. According to Burger and Scholz [48], female medical students showed significantly higher values for depressive symptoms than male medical students. However, gender itself did not appear as a significant predictor in our stepwise regression model. There has been extensive research on the matter of gender in mental health. However, we feel that a further discussion may be ethically challenging and could be futile in this essay. 

As expected, *neuroticism* was highly correlated with the BDI-II sum score. On the one hand, neuroticism and depression share genetic factors that predispose to both [49]. Further, person – situation interactions may influence the perception of a subject’s personality traits and state of character [50]. However, neuroticism was identified as the only personality trait that predicted non-specific psychological stress [51] and medical school stress [52]. Drake et al. [51] suggested that mindfulness had moderating effects on the relationship between neuroticism and psychological stress, hence interventions like these may be useful and should be the subject of further research. 

Results indicated that *insufficient emotional support* exhibits negative effects on depressive symptoms. This is in line with current research shows that emotional support has protective effects from depression [53], [54]. Yet as mentioned by Gariépy et al. [54], the source of social support most consistently associated with protection from depression was spousal support followed by support from family, friends and children. Most of the surveyed students (76.6%, see attachment 2 ) claimed to have *little time for partner, friends, family.*

Offering skilled emotional support through counselling may close the gap to *sufficient emotional support* for students. However, counselling demands at least a minimum of a subject’s personal time – something with which most students reported to struggle. This dilemma may be addressed by curricular changes that allow for a better integration of academic studies and social support. Additionally, speaking to a professional counsellor may be easier for affected students due to stigmatization concerns regarding their peers and families [55]. Furthermore, actively offering adequate counselling to affected medical students may illustrate a rejection of the stigma of mental ill health on the university’s part [56] and send a clear signal of support to their students. 

According to our data, the use of relaxation techniques was associated with lower levels of depressive symptoms. As suggested by Jorm et al. [57], relaxation techniques were more effective at reducing self-rated depressive symptoms than minimal or no treatment. They could be a way to bridge waiting times until the commencement of counselling. 

In general, students who are ill with Major Depression should be referred to skilled healthcare professionals. A university’s focus should be on the primary prevention and low-threshold aid for mildly to moderately burdened students. Especially for the latter, interventions and support are needed. Possible targeted intervention programs for burdened medical students have been discussed above. They should be subject of further research to evaluate their effects on depressive symptoms without exception. To identify the severely burdened students who are in need of professional medical help a regular, self-administered, self-report screening of depressive symptoms could be discussed. 

### Limitations of this study

Elevated symptom scores in questionnaires on depressive symptoms must not be confused with a higher prevalence of depressive illness such as Major Depression. A self-reporting instrument such as the BDI-II cannot replace an objective examination by professionals. Some argue that such instruments may report the prevalence of depressive symptoms inaccurately [58]. However, the BDI-II reports high consistency between its classification and a clinical diagnosis of depression. It also proved to differentiate well between different grades of depression and was sensitive to change [42]. While the BDI-II must not replace clinical diagnosis, it is a reliable tool to estimate the prevalence of depressive symptoms and depression burden beyond clinical symptomology. An empiric control for life events and other confounding factors of the depressive symptoms’ severity as well as control of cofactors associated with depressive symptoms should ultimately take place. Finally, targeted interventions should be subject of further research.

On another note, the cross-sectional setting used within this study may diminish the value of its results. Scholz [59] and Burger et al. [60] have done admirably in illustrating the continuous rise of depressive symptoms and the decline of mental quality of life among medical students during the first two years of study. However, studies evaluating depressive symptoms along with the entire duration of medical studies in terms of longitudinal surveys are needed. They also could disclose better insights into the students’ use of possible interventions and their efficiency. 

Another notable aspect regarding study design are the respective surveys that were being conducted at different times of a running academic term. This may limit the comparability of our results due to differences in stress levels of our subjects in effect to curricular differences (e.g. exams). Finally, it should be noted that the current study was conducted at one middle-sized German university, hence limiting its ability to be generalized onto other medical student populations. Further research should include multiple universities. 

## 5. Conclusions

To conclude, every fifth medical student surveyed, reported depressive symptoms of at least mild severity. The majority of the surveyed medical students felt unable to confide their worries to someone else and lamented not having enough time for social interaction with peers, family and hobbies. Neuroticism and insufficient emotional support were associated with depressive symptoms among medical students and may represent risk factors. Spending time with peers and family, intake of regular meals and confiding one’s worries to classmates were associated with lower levels of depressive symptoms. 

## Ethical significance of this research

Our results on predictive factors on depressive symptoms should not be used to assess potential medical students of their resilience towards depressive symptoms. 

## Acknowledgements

To the medical students who made this study possible - thank you for your patience. 

## Competing interests

The authors declare that they have no competing interests. 

## Supplementary Material

Self-description questionnaire

Correlations of potential risk factors and protective factors with BDI-II scores for subsamples

## Figures and Tables

**Table 1:**
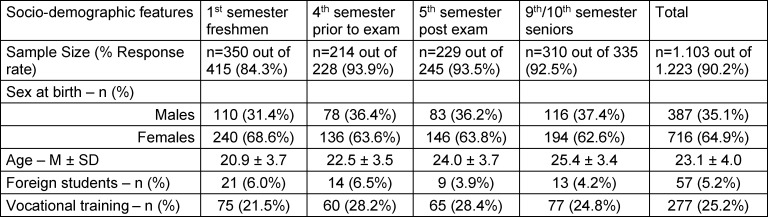
Sociodemographic characteristics

**Table 2:**
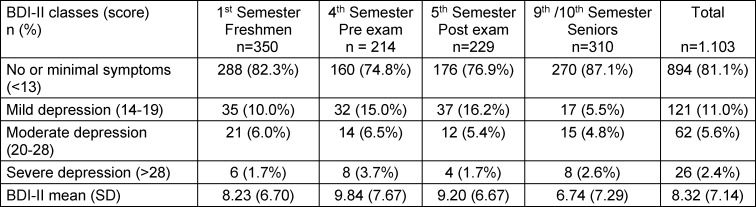
BDI-II scores of surveyed subsamples

**Table 3:**
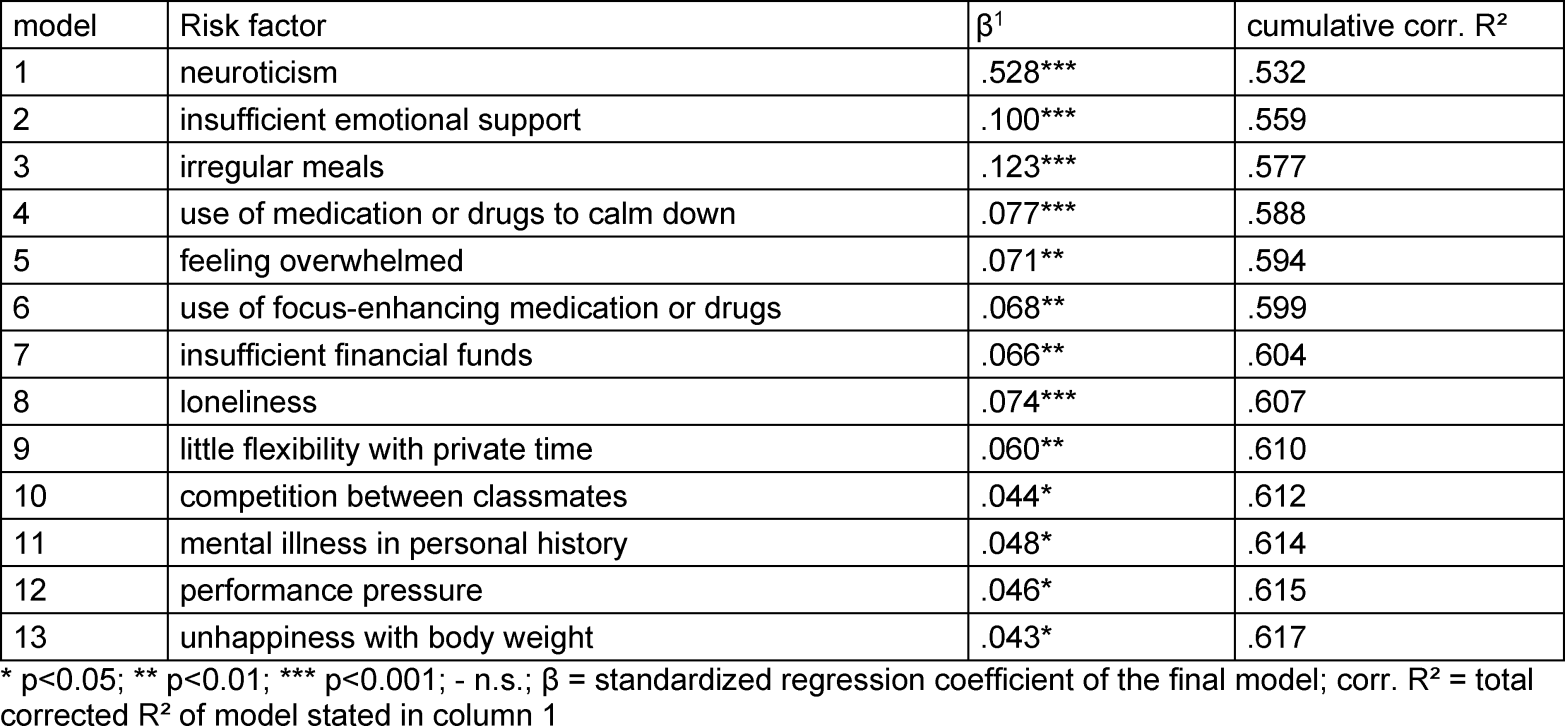
Stepwise linear regression model for prediction of BDI-II total score for the total study sample
