# Supplementary material for: Prevalence and predictive factors for depressive symptoms among medical students in Germany – a cross-sectional study
Source: GMS J Med Educ. 2022 Feb 15;39(1):Doc13. doi: 10.3205/zma001534 (PMC8953191; doi:10.3205/zma001534)
Supplement: Correlations of potential risk factors and protective factors with BDI-II scores for subsamples [file JME-39-13-s-002.pdf]

Attachment 2: Correlations of potential risk factors and protective factors with BDI-II scores for subsamples

Kendall-tau<br>(% of affected students<br>or median MD)	1st Semester<br>Freshmen<br>n = 350	4th Semester<br>Pre exam<br>n = 214	5th Semester<br>Post exam<br>n=229	9th /10th Semester<br>Seniors<br>n=310	Total<br><br>n=1.103
**Risk factors**					
neuroticism	.533***	.553***	.594**	.463***	.538***
age	-	-	-	.086*	-
being an international student	.089* (6.0%)	.124* (6.5%)	.124* (3.9%)	-	.088*** (5.2%)
mental illness in 1st degree relative	-	-	-	.106* (28.1%)	.088* (27.3%)
prior own mental health issues	.128** (12.1%)	.183*** (12.6%)	.183*** (17.5%)	.289*** (14.8%)	.195*** (14.1%)
loss or separation from parent	-	.115* (16.3%)	.129* (17.0%)	-	.052* (18.2%)
studies in order to gain respect	-	-	.196*** (7.8%)	-	.064* (6.9%)
studies due to family pressure	-	-	.123* (5.2%)	.106* (6.5%)	.050* (4.8%)
years until admission into studies	-.113* (16.2%)	.118* (22.8%)	-	-	-
insufficient financial funds	.163*** (20.3%)	.212*** (19.1%)	-	.230*** (18.7%)	.171*** (19.6%)
number of hours studying per week	-	.133** (MD=20)	-	.095* (MD=10)	.136*** (MD=15)
number of hours working per week	-	-	-	.119** (MD=4)	-
Subject experiences the following:					
uncertainty to graduate from studies	.119** (9.4%)	.268*** (8.8%)	.171** (3.5%)	.132** (1.3%)	.168*** (5.8%)
confiding own worries to no one	-	-	-	.133** (90.6%)	.054* (88.0%)
competition between classmates	.143*** (4.6%)	.247*** (12.6%)	.162*** (7.9%)	.154*** (5.5%)	.179*** (7.1%)
little flexibility with private time	-	.129* (31.2%)	-	.149*** (27.4%)	.110*** (24.5%)
feeling overwhelmed	.294*** (26.6%)	.226*** (26.0%)	.296*** (27.1%)	.220*** (10.6%)	.287*** (22.1%)
loneliness	.312*** (17.1%)	.196*** (13.0%)	.231*** (15.7%)	.173*** (7.1%)	.246*** (13.2%)

Attachment 2 to Pukas L, Rabkow N, Keuch L, Ehring E, Fuchs S, Stoevesandt D, Sapalidis A, Pelzer A, Rehnisch C, Watzke S. *Prevalence ans predictive factors for depressive symptoms among medical students in Germany – a cross-sectional study*. GMS J Med Educ. 2022;39(1):Doc13. DOI: 10.3205/zma001534

performance pressure	.221*** (43.3%)	.292*** (55.8%)	.308*** (48.0%)	.222*** (29.7%)	.278*** (42.9%)
insufficient emotional support	.328*** (17.4%)	.287*** (21.5%)	.239*** (16.7%)	.336*** (16.2%)	.304*** (17.7%)
lack of time for hobbies	-	.220*** (82.3%)	.124* (81.2%)	.254*** (63.2%)	.195*** (75.8%)
lack of time for partner, friends or family	.185*** (75.6%)	.210*** (82.3%)	.177*** (83.3%)	.228*** (68.0%)	.222*** (76.6%)
unhappiness with body weight	.206*** (39.8%)	.221*** (36.7%)	.200*** (33.2%)	.251*** (41.7%)	.203*** (41.7%)
Consequences:					
number of smoked cigarettes per day	.097* (MD=0)	-	.160*** (MD=0)	.122*** (MD=0)	.097*** (MD=0)
being questioned on alcohol use	-	-	-	-	.051* (8.4%)
use of caffeinated beverages	.188*** (44.2%)	-	-	.131*** (3.5%)	.126*** (54.7%)
use of focus-enhancing medication or drugs	-	.121* (6.5%)	.123* (2.2%)	.191*** (3.5%)	.129*** (2.9%)
use of food to calm down	.188*** (14.2%)	.198*** (16.7%)	-	.139*** (16.8%)	.158*** (16.3%)
use of medication or drugs to calm down	.211*** (4.8%)	.215*** (10.2%)	.198*** (6.9%)	.166*** (5.5%)	.199*** (6.5%)
confiding worries to a healthcare professional	.158*** (5.2)	.283*** (11.2%)	.173** (13.1%)	.177*** (14.8%)	.177*** (10.7%)
**Protective factors**					
assigned sex at birth: male	-.204*** (31.4%)	-	-	-	-.095*** (35.1%)
prior vocational training	-.090 (21.3%)	-	-	-	-
studying nearby own family	-.114* (19.6%)	-	-	-	-
nr. of hrs with partner, friends & family / week	-.124*** (MD=12)	-.173*** (MD=16)	-	-.124*** (MD=20)	-.134*** (MD=18)
nr. of hrs with hobbies & exercise / week	-.133*** (MD=5)	-.201*** (MD=6)	-.124*** (MD=7)	-.181***(MD=10)	-.179*** (MD=6)
using relaxation techniques	-	-	-	-.102* (21.6%)	-
regular meals	-.181*** (83.2%)	-.198*** (85.5%)	-.203*** (89.1%)	-.159*** (87.4%)	-.180*** (86.1%)
confiding worries to classmates	-	-	-.126* (36.2%)	-	-.067*** (31.2%)
